# Effects of Ectopic Expression of NGAL on Doxorubicin Sensitivity

**DOI:** 10.18632/oncotarget.691

**Published:** 2012-10-20

**Authors:** William H. Chappell, Stephen L. Abrams, Giuseppe Montalto, Melchiorre Cervello, Alberto M. Martelli, Saverio Candido, Massimo Libra, Jerry Polesel, Renato Talamini, Ralph Arlinghaus, Linda S. Steelman, James A. McCubrey

**Affiliations:** ^1^ Department of Microbiology & Immunology, Brody School of Medicine, East Carolina University, Greenville, NC, USA 27834; ^2^ Department of Internal Medicine and Specialties, University of Palermo, Palermo, Italy; ^3^ Consiglio Nazionale delle Ricerche, Istituto di Biomedicina e Immunologia Molecolare “Alberto Monroy”, Palermo, Italy; ^4^ Department of Biomedical and Neuromotor Sciences, Università di Bologna, Bologna, Italy; ^5^ Institute of Molecular Genetics, National Research Council-Rizzoli Orthopedic Institute, Bologna, Italy; ^6^ Department of Bio-Medical Sciences, University of Catania, Catania, Italy; ^7^ Unit of Epidemiology and Biostatistics, Centro di Riferimento Oncologico, IRCCS, Aviano, Italy; ^8^ Department of Molecular Pathology, MD Anderson Cancer Center, University of Texas, Houston, TX

**Keywords:** NGAL, Lcn2, Doxorubicin, lipocalins, siderocalins, iron transport, MMP-9, drug resistance

## Abstract

Neutrophil gelatinase-associated lipocalin (NGAL, a.k.a Lnc2) is a member of the lipocalin family which has diverse roles including stabilizing matrix metalloproteinase-9 from auto-degradation and as siderocalins which are important in the transport of iron. NGAL also has important biological functions involved in immunity and inflammation as well as responses to kidney damage. NGAL expression has also been associated with certain neoplasia and is important in the metastasis of breast cancer. Many advanced cancer patients have elevated levels of NGAL in their urine and it has been proposed that NGAL may be a prognostic indicator for certain cancers (e.g. breast, brain, and others). NGAL expression is detected in response to various chemotherapeutic drugs including doxorubicin and docetaxel. We were interested in the roles of NGAL expression in cancer and whether it is associated with chemotherapeutic drug resistance. In the present study, we investigated whether increased NGAL expression led to resistance to the chemotherapeutic drug doxorubicin in normal breast epithelial cells (MCF-10A), breast cancer cells (MCF-7), and colorectal cancer cells (HT-29). We infected the various cell lines with a retrovirus encoding NGAL which we constructed. Increased NGAL expression was readily detected in the NGAL-infected cells but not the empty vector-infected cells. However, increased NGAL expression did not alter the sensitivity of the cells to the chemotherapeutic drug doxorubicin. Thus, although NGAL expression is often detected after chemotherapeutic drug treatment, it by itself, does not lead to doxorubicin resistance.

## INTRODUCTION

Identifying the pathways activated and critical for cancer initiation and subsequent spread (invasion and metastasis) are essential for improved cancer therapy. Also, it is essential to understand how cells develop resistance to various therapeutic approaches and whether the resistance results from intrinsic or extrinsic events. Over the past 35 years, many genes have been identified which can cause or contribute to cancer [1,2]. These include two major classes of genes, the oncogenes and the tumor suppressor genes [3,4]. Moreover, microRNAs (miRNAs) and epigenetic modifications have been shown to be important in regulating cancer progression [5-8]. In some cases, the genetic culprit involved in a particular cancer may be known (e.g., BCR-ABL in chronic myeloid leukemia [9,10]. However, in most cases, there are multiple genetic and epigenetic events occuring which interact and develop into a cancer cell capable of becoming metastatic and/or drug resistant. In addition, there are other important contributions by the tumor microenvironment which aid in the progression of the cancer as well as resistance to various therapeutic approaches and the survival of cancer initiating cells [11-13].

One factor which may be important for cancer survival and metastasis is neutrophil gelatinase-associated lipocalin (NGAL). One of the genomic responses to common cancer treatments such as radiation and chemotherapy is the induction of NGAL expression [14-19]. NGAL may act to stabilize MMP-9 and increase its ability to degrade the extracellular matrix, hence promoting metastasis.

NGAL expression is regulated by the transcription factors: NF-kappaB, CEBP and others [20-24]. Radiation and chemotherapy may induce reactive oxygen species (ROS) that result in NF-kappaB activation [25,26] and subsequent downstream NGAL transcription. In addition, the tumor microevironment may alter NF-kappaB activity [27]. Chemo- and radiotherapy could result in the synthesis of NGAL in cancer cells which may lead to the development of therapy-resistant cells. These cells can contribute to the reemergence and metastasis of the cancer as increased NGAL expression may allow the cells to persist under conditions where the therapy-sensitive cancer cells could not normally survive.

Cancer cells have increased demands for intracellular iron. NGAL is a member of the lipocalin family and as such is capable of serving as siderocalins or molecules involved in the transport of iron and other molecules [28]. Iron is essential for many key processes, including the rate-limiting step in DNA synthesis carried out by ribonucleotide reductase [29]. Iron (Fe^++^) is also required for cells to progress through the cell cycle from G_1_ to S phase. Tumor cells have a high requirement for iron and express elevated levels of the transferrin receptor-1 [30-34]. Novel chelators of iron are being considered for cancer treatment [35]. Iron chelators, such as Desferrioxamine (DFO), inhibit cellular iron transport and have been evualated in various cancer clinical trials [36]. Oxygen and iron concentrations may be altered in the tumor microenvironment due to drastic tumor growth [37-40]. In order for a cancer cell to survive, invade, and metastasize it may have to have increased iron transport as well as elevated glycolysis [11-14,40,41]. The role of iron transport in chemotherapeutic drug resistance of cancer cells is complex and may depend on the particular drug and cancer type investigated [42,43]. Interestingly, some iron depletors have been shown to decrease resistance of certain cancer cells to chemotherapeutic drugs including doxorubicin [44,45].

Increased levels of NGAL have been detected in the urine of patients with various types of cancer (i.e. brain, breast, colon, ovarian, pancreatic and prostate). Novel non-invasive urine-based tests could prove useful for the detection and/or prognosis of many cancer types [46-49].

The role(s) of NGAL in chemotherapeutic drug resistance, invasion, and cancer metastasis are not fully elucidated [15]. Targeting NGAL could result in decreased cancer cell survival and tumor regression as well as improve the effectiveness of radiation and chemotherapy in cancer therapy. NGAL is considered by some scientists to have properties of an oncogene. It has been shown in some studies to increase the mobility, invasion, metastasis, and tumorigenesis of certain cancer cells [breast, colorectal cancer (CRC)] [50-52]. Elevated expression of NGAL increases the invasiveness of certain cancer cells, while inhibition of NGAL expression decreases their invasiveness and metastasis [17, 19, 50]. New approaches to target MMP-9/NGAL are needed as MMP-9 inhibitors have not performed well in clinical cancer trials [54] and NGAL has functions which are independent of MMP-9.

**Figure 1:**
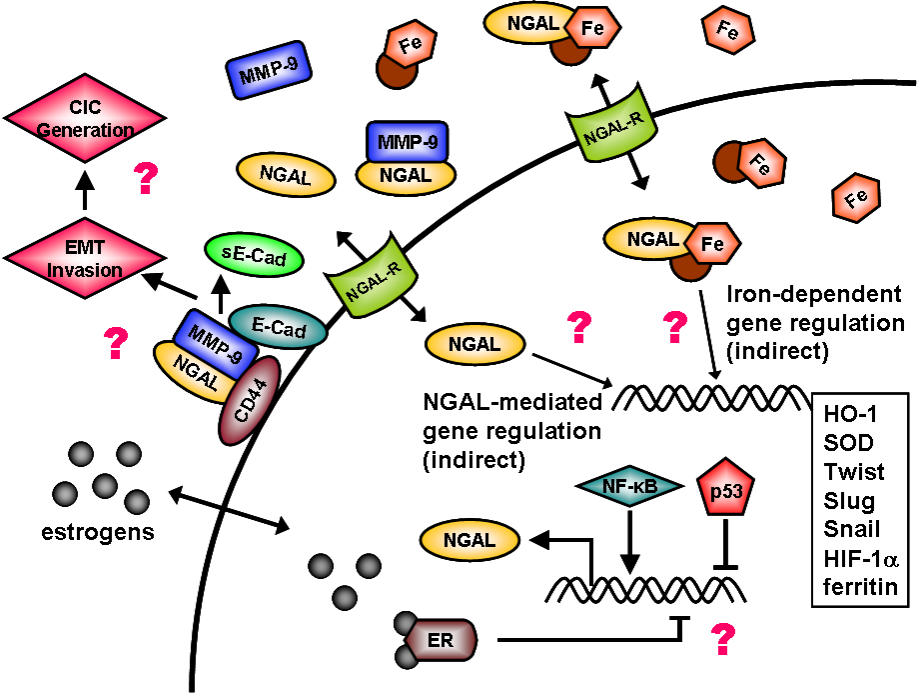
Overview of Potential Effects of NGAL on Cellular Growth NGAL may serve diverse biochemical functions from stabilizing MMP-9 at the cellular membrane in association with CD44 and E-Cadherin (E-Cad), to roles in promoting EMT and invasion. In addition, NGAL may have roles in the transport of iron into the cell and influence iron-mediated gene expression. NGAL gene expression is infuenced by many transcription figures including NF-κB, p53, and potential hormones such as the estrogen receptor (ER). Iron mediated gene expression is complex and can influence the expression of many genes involved in cancer metastasis such as Twist, Slug, and Snail.

NGAL may exert many different effects that are important in invasion and metastasis. NGAL can stabilize MMP-9 at the cell surface [55-57]. This complex may be associated with CD44 which may promote the cleavage of E-Cadherin (E-Cad) into soluble (s)E-Cad thereby promoting EMT [57,58]. A diagram illustrating potential effects of NGAL on cell survival is presented in Figure 1. In the following studies, we examined the effects of ectopic NGAL expression on the sensitivity of breast cancer and CRC to a common chemotherapeutic drug used to treat many cancer patients (doxorubicin, a.k.a Adriamycin). We chose to examine the effects of ecotopic NGAL expression on sensitivity to doxorubicin on two different different types of cancers. Breast cancer which is generally sensitive to doxorubicin therapy and CRC which is considered resistant to doxorubicin therapy. An immortalized breast epithelial cell line, MCF-10A, was also examined in these studies.

## RESULTS

### Infection of Cell Lines with a Retrovirus Encoding NGAL and Expression of Ectopic NGAL

**Figure 2:**
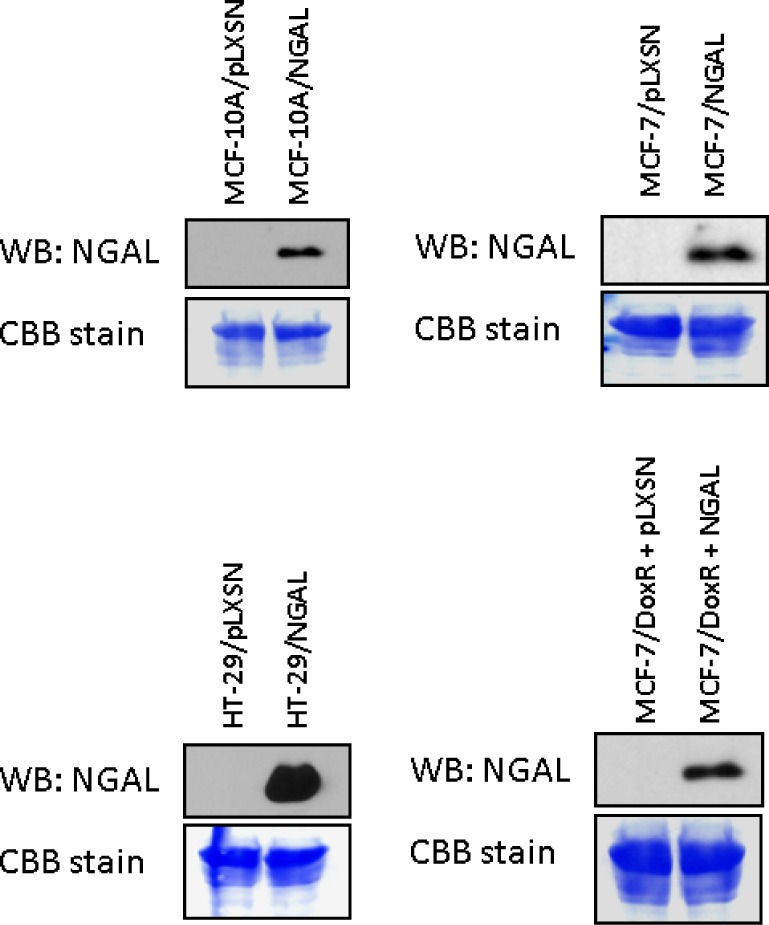
NGAL Expression in pLXSN and NGAL Infected Cells pLXSN empty vector and NGAL infected MCF-10A, MCF-7, and HT-29T were cultured for 24 hours in the absence of FBS and then the supernatants were analyzed for the expression of NGAL as described in Material & Methods. All the experiments in this figure were performed at the same time (set up on the same day).

MCF-10A, MCF-7, and HT-29 cells were infected with a retrovirus encoding NGAL or the empty retrovirus pLXSN. NGAL was detected in the supernatants from NGAL retrovirus infected cells (Figure 2). In contrast, NGAL was not detected in the empty vector pLXSN virus infected cells.

### Effects of Enforced NGAL Expression on Sensitivity to Doxorubicin

**Figure 3:**
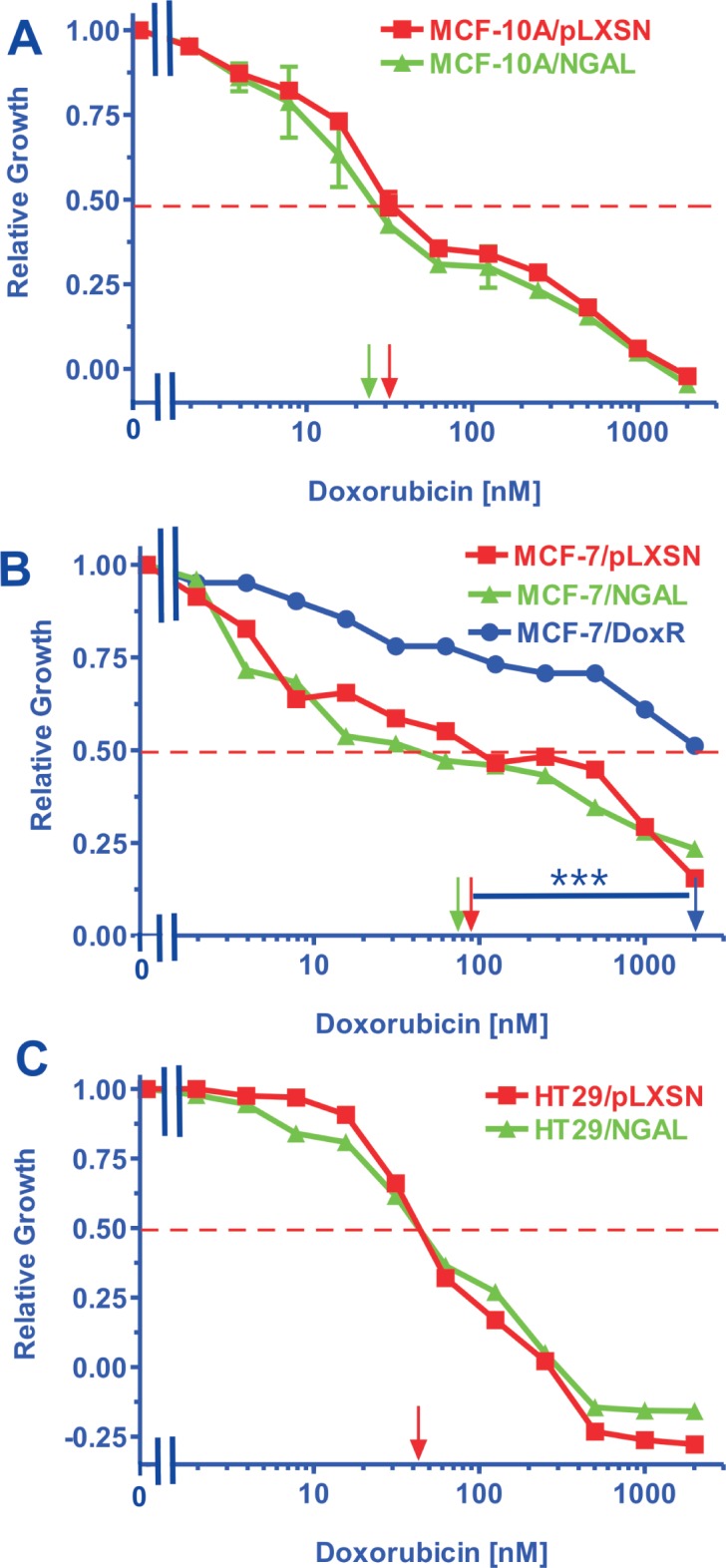
Doxorubicin IC_50_ analysis in NGAL and pLXSN Infected Cells and Doxorubicin-Resistant MCF-7/Dox^R^ Cells Cells were collected and seeded (2000 cells/well) in 96 well plates. The following day, serial two-fold dilutions of doxorubicin were added to the wells. Four days later, MTT assays were performed to determine the effects of doxorubicin on: Panel A) MCF-10A/pLXSN (solid squares), MCF-10A/NGAL (solid triangles), Panel B) MCF-7/pLXSN (solid squares), MCF-7/NGAL (solid triangles) and 25 nM doxorubicin selected MCF-7/Dox^R^ cells (solid circles), Panel C) HT-29/pLXSN (solid squares), HT-29/NGAL (solid triangles). A hatched horizontal line is present at the 50% relative growth mark from which the IC_50_ can be calculated. A veritical arrow indicates the IC_50_. The statistical significance was determined by the unpaired *t* test (***, P<0.001). All the experiments in this figure were performed at the same time (set up on the same day). These experiments were repeated multiple times and similar results were obtained.

We next examined the effects of elevated NGAL expression on the sensitivity of the cell lines to doxorubicin using MTT assays. A representative MTT assay is presented in Figure 3. Also included in these anaylses were the effects of doxorubicin on a doxorubicin-resistant MCF-7 line named MCF-7/Dox^R^ (Chappell AER paper in press) that were performed at the same time. Clearly, the doxorubicin-resistant MCF-7/Dox^R^ is approximately 20-fold more resistant to doxorubicin than the MCF-7/pLXSN or MCF-7/NGAL cell lines demonstrating that NGAL expression alone does not confer doxorubicin resistance in these cells.

The doxorubicin IC_50_s for various NGAL- and pLXSN-infected cell lines representing different tissues are presented in Table 1. Increased NGAL expression did not significantly (>2-fold) alter the IC_50_s for doxorubicin in the cell lines examined.

**Table 1:** Effects of Ectopic NGAL Expression on the Doxorubicin IC50 in Cell Lines Derived from Different Cancer Types^1^

Cell Line	Type	Doxorubicin IC50
MCF-10A/pLXSN	mammary luminal epithelial, non malignant, near diploid	32 nM
MCF-10A/NGAL		30 nM
MCF-7/pLXSN	breast cancer ER+, luminal	90 nM
MCF-7/NGAL		80 nM
MCF-7/DoxR	doxorubicin resistant MCF-7	2000 nM
HT-27/pLXSN	colorectal cancer	40 nM
HT-27/NGAL		40 nM

1 MTT analysis was performed with different unselected cancer lines and certain 25 nM doxorubicin resistant (DoxR). Determined by plating 5,000 cells/well in 96 well plates in phenol red free RPMI 1640 + 10% FBS. Serial 2-fold dilutions (n=12 dilutions) of doxorubicin were dispensed into 8 wells per each doxorubicin concentration after the first day. MTT analysis was performed after 4 additional days of incubation and results were normalized to untreated cells as described [62].

## DISCUSSION

These studies were undertaken to determine whether increased NGAL expression altered the sensitivity to the chemotherapeutic drug doxorubicin in cancer cell types which are normally sensitive to doxorubicin (breast) and cancer cell types which are normally resistant to doxorubicin (CRC) as well as immortalized breast epithelial cells (MCF-10A) which are not malignant. NGAL may have roles in iron transport which may be associated with chemoresistance in certain cancers. Some studies have shown that iron chelators will reduce chemotherapeutic drug resistance [44,45]. However, in our studies, elevated NGAL expression did not alter the sensitivity to doxorubicin of neither the breast cancer nor CRC cells examined. Additionally, it did not alter the sensitivity of the immortalized breast epithelial MCF-10A cells. Note, it is also conceivable that elevated NGAL expression could have made the cells more sensitive to doxorubicin, but that did not appear to occur in these cells, or other cells examined.

The doxorubicin resistant MCF-7/Dox^R^ cell line was also infected with the retrovirus encoding NGAL (Figure 2), however, these cells were not more resistant to doxorubicin than empty vector control MCF-7/Dox^R^ cells (data not presented). NGAL expression was not detected at higher levels in drug resistant MCF-7/Dox^R^+pLXSN cells than MCF-7/pLXSN (Figure 2). Moreover, the doxorubicin resistant MCF-7/Dox^R^ cells do not normally express NGAL; however, upon treatment with doxorubicin increased NGAL protein has been detected [59]. Elevated NGAL expression does not appear, by itself, to increase the resistance to doxorubicin in the cells examined. Such changes could be occurring in the regulation of various signaling pathways or drug transporters in these cells.

Although NGAL expression has been associated with a poor prognosis in breast and other cancers [24,60,61], elevated expression of NGAL does not alter the IC_50_ for the chemotherapeutic drug doxorubicin. Furthermore, elevated expression of NGAL did not alter the responses of either the CRC line HT-29 or the immortalized epithelial line MCF-10A to doxorubicin.

## MATERIALS & METHODS

### Cell Lines and Growth Factors

The breast cancer (MCF-7) and CRC (HT-29) cells lines were obtained from the ATCC (Rockville, MD, USA). Cells were maintained in a humidified 5% CO_2_ incubator at 37^°^C with RPMI-1640 [(RPMI) Invitrogen, Carlsbad, CA, USA] supplemented with 10% fetal bovine serum (FBS) (Atlanta Biologicals, Atlanta, GA, USA). This complete RPMI media is abbreviated cRPMI. The immortalized breast epithelial MCF-10A line was obtained from the ATCC and cultured in DMEM/F12 (Invitrogen) medium containing: 2.5mM L-glutamine, supplemented with 5% heat inactivated equine serum (Invitrogen), 500 ng/ml hydrocortisone (Sigma-Aldrich), 21.5 ng/ml epidermal growth factor (EGF) (Sigma-Aldrich), 10 μg/ml insulin (Sigma-Aldrich), 100 ng/ml cholera toxin (Sigma-Aldrich), and 15mM HEPES (Sigma-Aldrich).

### Methylthiazol Tetrazolium Assay

Methylthiazol tetrazolium (MTT) assays were performed to determine a cell line's sensitivity to chemotherapeutic drugs. 2 × 10^3^ cells per well were plated in 96-well plates in 100 μL of cRPMI without phenol red (Invitrogen™, Carlsbad, CA) and allowed to attach overnight under normal culture conditions. The next day, serial two-fold dilutions of a chemotherapeutic drug were made and 100 μL of each dilution were added to a corresponding well on the 96-well plate. Cells were incubated for four days under normal culture conditions. On the fourth day, 22.2 μL of a 5 mg/mL solution of thiazolyl blue tetrazolium bromide (Sigma-Aldrich, Saint Louis, MO) in 1X PBS was added to each well and incubated for 90 minutes at 37^°^C. The media was then removed and 150 μL of dimethyl sulfoxide (DMSO) (Fisher Scientific, Pittsburgh, PA) was added to resuspend formazin crystals to produce a purple color which was subsequently read on a Multiskan EX Microplate photometer (Thermo Scientific, Hudson, NH) at a wavelength of 570 nm. Colormetric readings were normalized against plates of non-treated cells under identical culture conditions. Relative growth was calculated by dividing normalized cell growth values in the presence of drugs by normalized cell growth values in the absence of drugs and the results were graphed. Drug concentrations that killed at least 50% of the cells (IC_50_) were determined from the calculated graphed values [62].

### Construction of Retroviral Vector Containing NGAL

The pLXSN/NGAL retroviral expression vector (referred to here and throughout this study as NGAL) was constructed using the retroviral vector pLXSN (Clontech^©^, Mountain View, CA) as the backbone. The full length cDNA of human NGAL (*Lcn2*, GenBank accession no. BC033089) was amplified from the pcDNA3.1(+)-NGAL plasmid [51] using specific primers for regions flanking either ends of the gene insert. The PCR product was then inserted into a pCR2.1-TOPOR TA vector (Invitrogen^™^, Carlsbad, CA), subsequently digested with EcoRI endonuclease (New England Biolabs^®^, Ipswich, MA), and the resulting gene fragment was ligated into the EcoRI digested pLXSN vector.

### Packaging of Retroviral Vectors

Packaging of the retroviral vectors used in this study was as follows. 10 mL of a 0.1 % gelatin/sterile water solution (Specialty Media, Chemicon/Millipore^™^, Billerica, MA) was used to gelatin coat Corning 75 cm^2^ flasks (Corning, NY) for one hour at room temperature and then removed. Following two subsequent washes with 1X phosphate buffered saline (PBS) (110 mM NaCl, 2.1 mM KCl, 1.1 mM KH_2_PO_4_, 6.7 mM Na_3_PO_4_, pH 7.4), 6 × 10^6^ total 293T cells were plated in 10 mL cDMEM and allowed to adhere at 37^°^C overnight to the gelatin coated flasks. The next day, the culture media was removed and replaced with 10 mL fresh cDMEM one hour prior to transfection. DNA calcium phosphate precipitation was used to transfect 293T cells. 15 μg of the packaging vector pCL-Ampho (Imgenex^©^, San Diego, CA) was mixed with 35 μg of pLXSN/NGAL, in 1.5 mL of 0.25 M CaCl_2_. This mixture was then added drop wise to 1.5 mL of 2X HBS (50 mM HEPES, 280 mM NaCl_2_, 1.5 mM Na_2_HPO4, pH 7.17) and allowed to precipitate at room temperature for 45 minutes. After the precipitate incubation time, the solution of calcium-phosphate/DNA was added to the T75 flask of 293T cells and incubated for six hours at 37^°^C in a humidified incubator with 5% CO_2_. Media was then removed and replaced with 9 mL of a 15% glycerol/PBS solution for one minute at room temperature. This solution was then removed and the cells gently washed once with 1X PBS before adding back 10 mL of fresh cDMEM. Spent media containing the packaged virus particles were harvested 48 and 72 hours later. The viral supernatant was filtered through a 0.45 μm PVDF filter (Millipore^™^,Billerica, MA) to remove cellular debris, aliquoted, and used fresh for subsequent retroviral transductions or stored frozen at −80^°^C for later use. The viral supernatants were used to infected the various cell lines and stable transformed pools or either NGAL or pLXSN (empty vector control) were selected in media contain 2 mg/ml G418 (Geneticin^®^, Sigma-Aldrich) after 2-3 weeks of section. Medium was changed every 3 days with fresh medium containing 2 mg/ml G418.

### Detection of Secreted NGAL

Supernatant lysates used to determine the levels of NGAL protein in the supernatant were harvested and prepared as follows. All cell lines were plated in 6-well plates and cultured under normal culture conditions in cRPMI. Once the cells reached 80% confluency the media were removed, the cells were washed twice with 1X PBS, and 2 mL of fresh RPMI without FBS was added back. After 24 hours, the supernatants were harvested, cleared of cellular debris via centrifugation, and processed. Total protein from the collected supernatants was isolated by mixing 1 mL of the sample with 250 μL of 100% (w/v) trichloroacetic acid (TCA) (Sigma-Aldridge, Saint Louis, MO) and incubating the mixture at 4^°^C for 15 minutes. The sample pellets were collected via centrifugation at 14000 RPM for 15 minutes. The pellets were washed twice with ice cold 100% acetone and allowed to air dry. The protein pellets were resuspended in 25 μL of western sample buffer, boiled for 5 minutes, and examined by western blot analysis.

20 μL of the prepared supernatant samples was separated on 12% Tris-glycine poly-acrylamide gels and transferred to polyvinylidene fluoride (PVDF) membranes (Thermo Scientific, Rockford, IL). All membranes were blocked in 1X Tris-buffered saline + Tween-20 (TBST) (32 mM Tris, 125 mM NaCl, 0.5% Tween-20, pH 8) containing 1% bovine serum albumin (BSA) (Fisher/Thermo Scientific, Rockford, IL) overnight at 4^°^C with gently shaking. Blocked membranes were placed in appropriate dilutions of the NGAL antibody [50] in 1x TBST + 1% BSA overnight at 4^°^C with gentle shaking. The next day, membranes were washed four times with 1X TBST before adding the appropriate secondary antibody made up in 1X TBST plus 5% non-fat dry milk for one hour. After another round of four washes, the membranes were treated with horseradish peroxidase enhanced chemiluminescence (Pierce/Thermo Scientific, Rockford, IL) as per manufacturer's instructions and protein bands visualized via exposure to X-ray film (Research Products 42 International, Mount Prospect, IL). 0.1% coomassie brilliant blue R250 (CBB) (Sigma-Aldrich) in a 50% methanol/7% acetic acid solution was used to stain total protein on the cellular supernatant immunoblots as a loading control.
